# Primary hepatic adenosquamous carcinoma: a case report and review of the literature

**DOI:** 10.3389/fonc.2023.1328886

**Published:** 2023-12-15

**Authors:** Haidong Ai, Ting Gong, Yongbiao Ma, Guixu Ma, Jingjing Zhao, Xuelin Zhao

**Affiliations:** ^1^ Hepatobiliary and Pancreatic Medical Center, The First Affiliated Hospital of Weifang Medical College (Weifang People’s Hospital), Weifang, China; ^2^ Department of Ophthalmology, The First Affiliated Hospital of Weifang Medical College (Weifang People’s Hospital), Weifang, China

**Keywords:** primary adenosquamous carcinoma, hepatic tumor, radical surgical resection, adjuvant chemotherapy, immunohistochemistry, case report

## Abstract

Primary hepatic adenosquamous carcinoma is considered a rare subtype of intrahepatic cholangiocarcinoma, with fewer than 100 domestic and international cases reported. This malignancy exhibits a high degree of malignancy, strong invasiveness, and an unfavorable prognosis due to its propensity for early lymph node and intrahepatic metastasis. The etiology of this disease remains uncertain, and preoperative diagnosis is exceedingly challenging owing to the nonspecific clinical features and lack of specificity in imaging studies. Radical surgical resection is the most effective treatment for non-metastatic tumors, while targeted adjuvant therapy administered postoperatively can enhance therapeutic efficacy and delay tumor recurrence. This article documents the diagnostic and therapeutic course of a case of primary hepatic adenosquamous carcinoma treated at our medical institution, along with a comprehensive synthesis of the clinical characteristics and advances in the diagnosis and treatment of this disease, aiming to augment understanding and serve as a reference for future clinical endeavors.

## Introduction

Primary hepatic adenosquamous carcinoma (ASC) refers to a malignant liver tumor that simultaneously contains components of both adenocarcinoma (AC) and squamous cell carcinoma (SCC). It exhibits high invasiveness and poor prognosis, with an average survival time of less than 1 year. The 5th edition of the WHO classification of digestive system tumors categorizes ASC as a subtype of intrahepatic cholangiocarcinoma (ICC), which is clinically rare, accounting for only 2%-3% of all ICC cases. To date, there have been fewer than 100 reported cases worldwide (1, 2). Currently, there is no multicenter study with a large sample size or standardized guidelines for the diagnosis and treatment of this disease. This article aims to present a case report on the diagnosis and management process of primary hepatic ASC, along with a summary of the latest research progress both domestically and internationally, in order to enhance our understanding and experience in treating this condition. The following is a detailed account of the case.

## Case presentation

**Figure 1:**
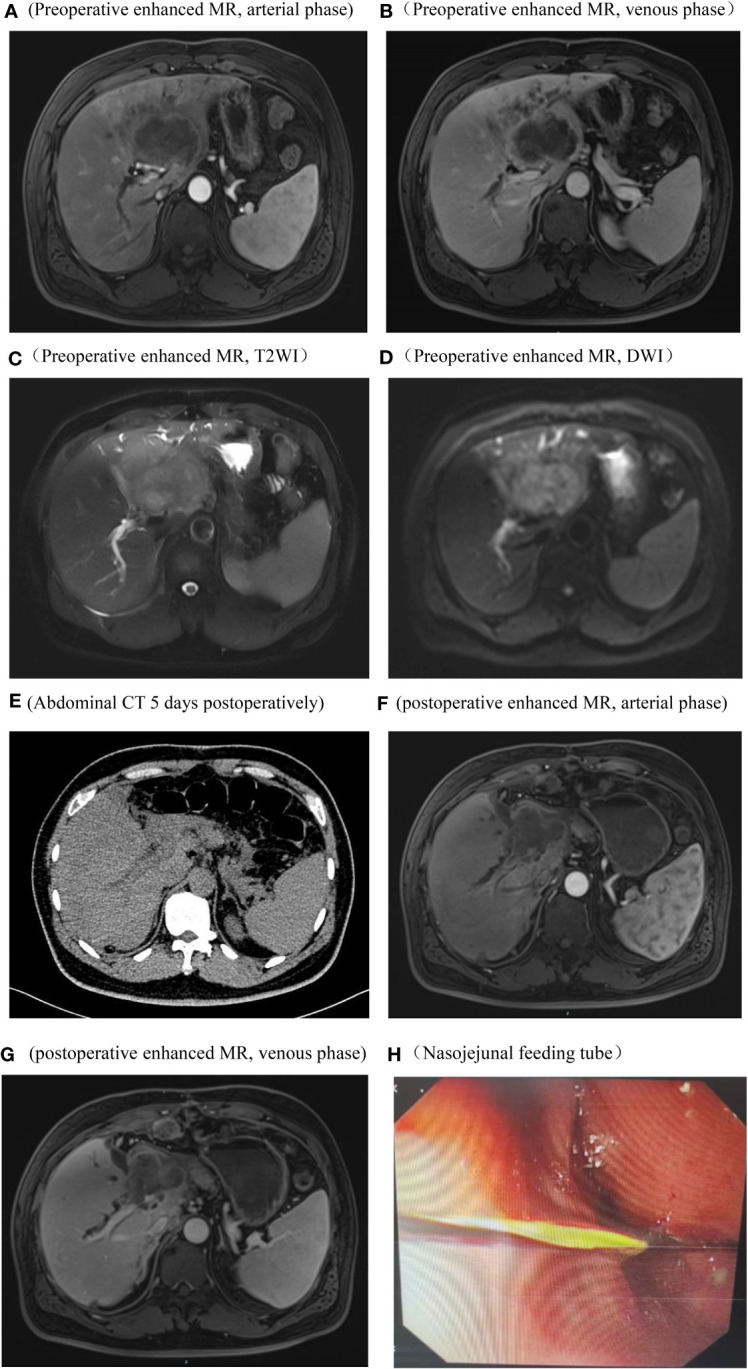
Imaging and endoscopic pictures. Preoperative abdominal enhanced MR, arterial phase, The tumor does not exhibit significant enhancement and shows lower signal intensity compared to the surrounding hepatic parenchyma **(A)** Preoperative abdominal enhanced MR, venous phase, the tumor periphery exhibits mild enhancement, while the central region demonstrates significantly lower signal intensity compared to the surrounding hepatic parenchyma **(B)** Preoperative abdominal enhanced MR, T2WI, the tumor demonstrates a long T2 signal, with signal intensity higher than the surrounding hepatic parenchyma **(C)** Preoperative abdominal enhanced MR, DWI, the tumor exhibits restricted diffusion **(D)** Abdominal CT on the 5th day postoperative, no significant fluid accumulation observed in the hepatic periphery or intra-abdominal cavity **(E)** Abdominal enhanced MR in the arterial phase and venous phases, 3 months postoperatively, Imaging reveals an irregular soft tissue mass in the hepatic hilum area following left hepatectomy. Enhanced scanning shows mild, uneven enhancement. Blurring is observed in the hepatic periphery, peritoneum, and abdominal fat interspace, with multiple nodules displaying heterogeneous enhancement within **(F, G)** Endoscopic placement of a nasojejunal nutritional tube **(H)**.

A 48-year-old male presented at The First Affiliated Hospital of Weifang Medical College on July 22, 2019, with a complaint of “persistent upper abdominal pain for over one month” He has a history of good physical health, with no known psychological disorders or family history of hereditary diseases. No relevant treatment was administered before admission. Laboratory results revealed the following: alanine aminotransferase (ALT) level (107 U/L, normal range 0-50 U/L), aspartate aminotransferase (AST) level (50 U/L, normal range 0-40 U/L), total bilirubin (TBIL) level (12 umol/L, normal range 0-23 umol/L), alkaline phosphatase (ALP) level (550 U/L, normal range 45-125 U/L), gamma-glutamyltransferase (GGT) level (364 U/L, normal range 4-60 U/L), and carbohydrate antigen 19-9 (CA19-9) level (1839.13 U/L, normal range 0-37 U/L). Other laboratory tests showed no significant abnormalities. Following admission, an abdominal enhanced magnetic resonance imaging (MRI) indicated a mass in the left lobe of the liver, intrahepatic bile duct dilation, and enlarged surrounding lymph nodes (**Figures 1A–D**). Based on our preoperative assessment, we diagnosed the patient with a primary malignant liver tumor, where intrahepatic cholangiocarcinoma (ICC) appears to be the most likely possibility, although other rare types of malignant liver tumors cannot be ruled out. Preoperative imaging studies indicated the lesion appears in a very close relationship with the right anterior pedicle, and enlarged regional lymph nodes. The tumor exhibits a high degree of invasiveness, making curative surgery challenging and carrying a heightened risk of recurrence. The patient and their family have been adequately informed of the surgical risks, including the potential inability to achieve an R0 resection, as well as the possibility and necessity of postoperative adjuvant chemotherapy, to which the family has expressed understanding and consent to the proposed treatment plan.

**Figure 2:**
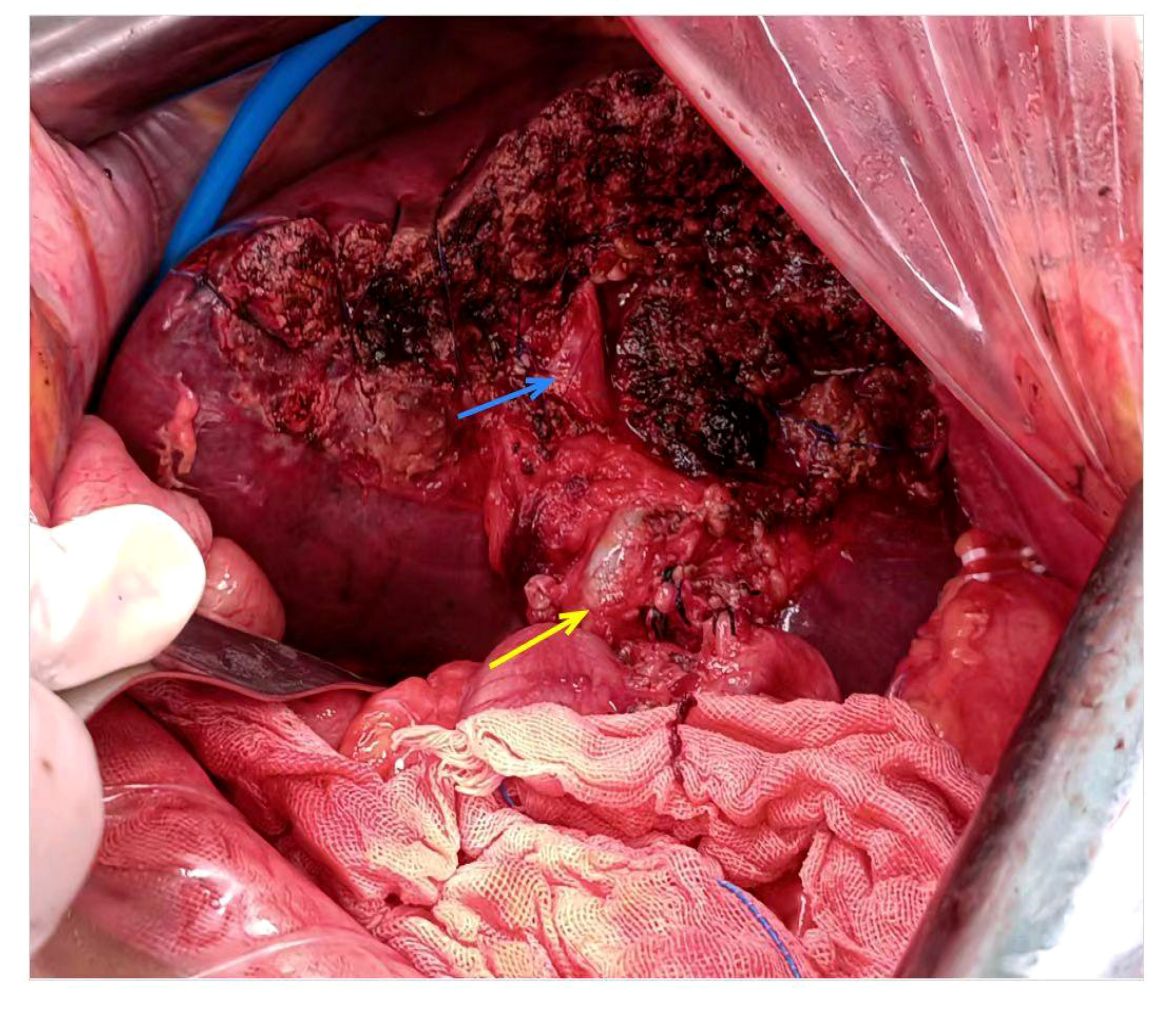
Intraoperative pictures. The blue arrow designates right anterior branch of the portal vein, the yellow arrow denotes common bile duct.

**Figure 3:**
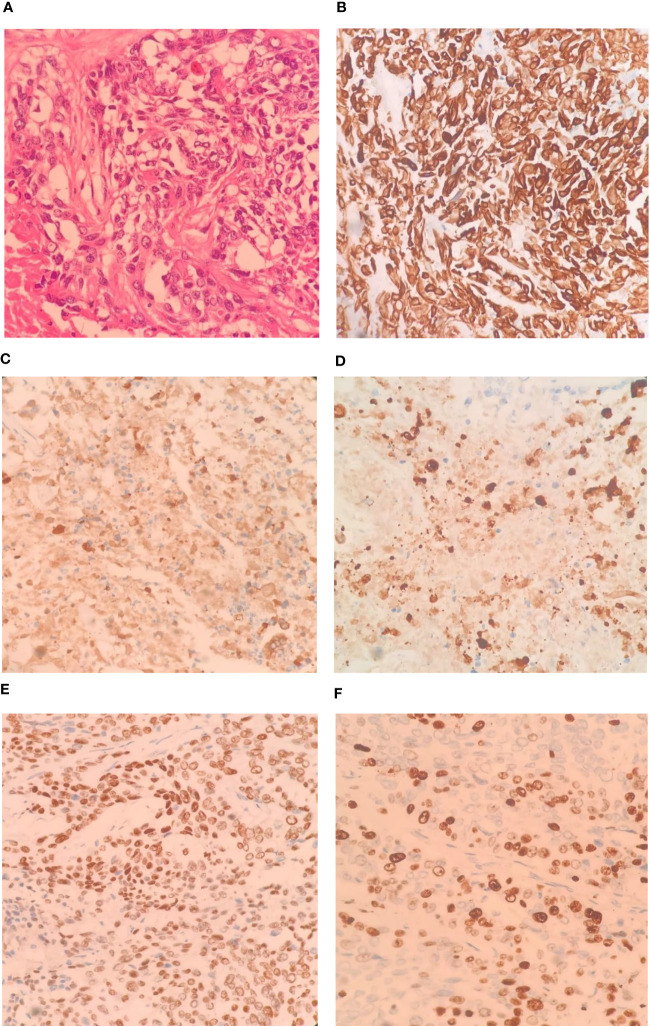
Pathological images and immunohistochemistry staining were conducted as follows: Tumor tissue sections stained (HE ×400), The tumor cells exhibit a pattern of solid nests, glandular formations, and reticular arrangements. Some of the nest formations show central keratinization. The cells possess abundant cytoplasm, large and deeply stained nuclei, and notable cellular atypia (**A**, ×400); CK-7 immunohistochemical staining exhibited diffuse positivity (**B**, ×400); CK-19 immunohistochemical staining exhibited diffuse positivity (**C**, ×400); CK-20 immunohistochemical staining displayed diffuse positivity (**D**, ×400); P40 immunohistochemical staining showed partial positivity (**E**, ×400); Ki-67 positive index is approximately 70%. (**F**, ×400).

The patient underwent left hemihepatectomy, cholecystectomy, and hepatic pedicle lymph node dissection. The operative procedure was as follows: Intraoperative exploration revealed a tumor located in the left half of the liver, compressing the first hepatic hilum. Multiple enlarged lymph nodes were observed in the hepatic pedicle region, with tumor invasion into the cystic duct and adjacent right anterior hepatic duct. Careful dissection of adhesions between the tumor and the right anterior hepatic pedicle was performed while preserving the right anterior hepatic duct. The hepatic hilum was dissected, and a skeletonized lymph node dissection was performed on the hepatic pedicle. The left hepatic artery and left branch of the portal vein were ligated and divided, ensuring complete removal of the tumor and left half of the liver. The surgery lasted for 3.5 hours, with an estimated intraoperative blood loss of approximately 100ml (**Figure 2**). Postoperative pathological examination revealed the tumor involving the capsule, vascular and neural invasion. The cut surface of the liver was clean, showing no residual tumor tissue. Lymph node metastasis was observed in the hepatic pedicle lymph nodes. Microscopic examination (**Figures 3A–F**) revealed tumor cells exhibiting both nest-like and glandular structures. Based on the results of immunohistochemistry, the diagnosis was determined to be primary hepatic ASC (with approximately 40% being moderately differentiated AC and 60% being poorly differentiated SCC). Immunohistochemical staining showed that CK7 (+), CK19(+), CK20(+), P40 partial (+), and the Ki-67 proliferation index of 70%.

**Figure 4:**
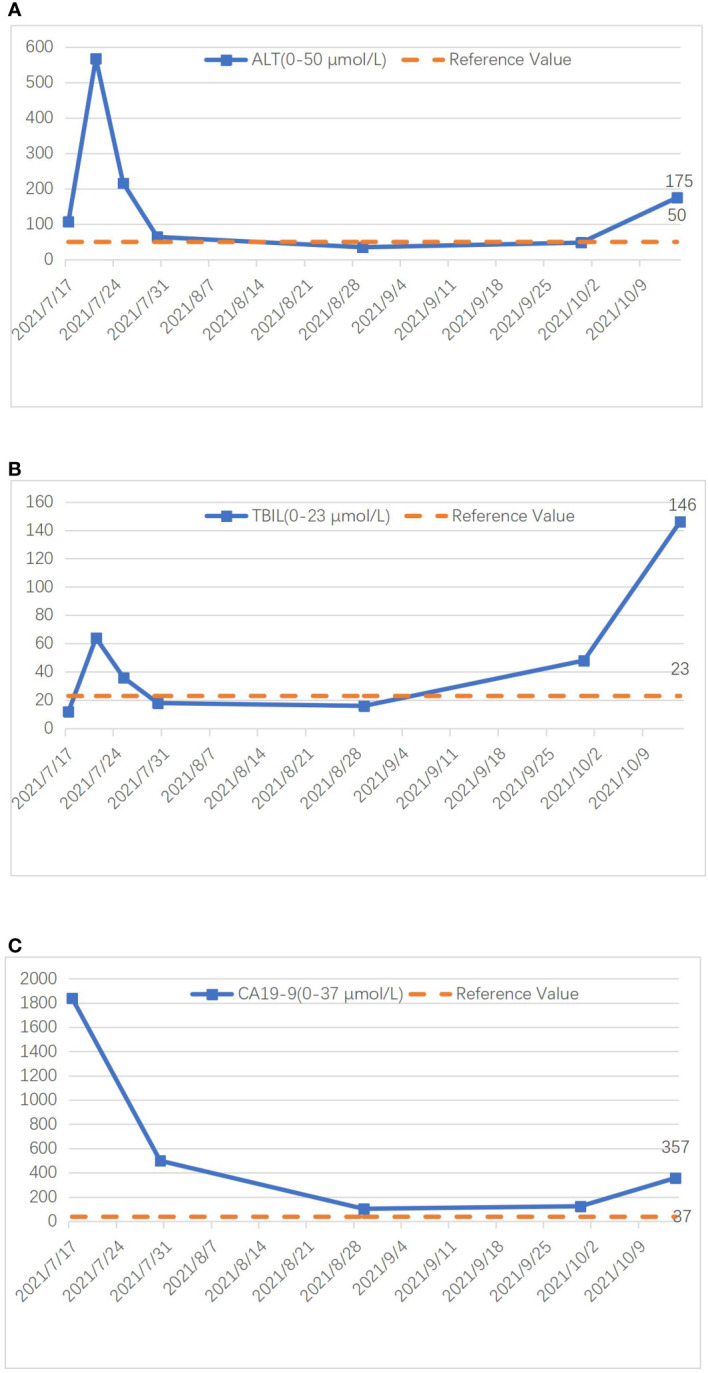
Graph illustrating the changing trends of ALT **(A)**, TBIL **(B)**, and CA19-9 **(C)** during treatment.

Preoperatively, we ruled out esophageal cancer, nasopharyngeal cancer, lung cancer, and gastric cancer, which are common sites for SCC. Combining the pathological findings, the definitive diagnosis was primary hepatic ASC. The patient, despite undergoing curative surgical resection, considering the high malignancy of hepatic ASC and its strong invasiveness, postoperative pathology indicated lymph node metastasis at the hepatic hilum. The efficacy of the sole surgical treatment was suboptimal. After the procedure, comprehensive discussions with the patient and their family were held regarding the disease, recommending postoperative systemic chemotherapy or hepatic arterial infusion chemotherapy (HAIC), with the possibility of adjunctive radiotherapy, and duly informing them of potential adverse reactions associated with these treatments. Regrettably, the patient declined any adjunctive therapy postoperatively. On the 5th day post-surgery, an abdominal CT scan revealed no significant peritoneal or perihepatic fluid accumulation (**Figure 1E**). AST and TBIL gradually returned to normal levels, while serum CA19-9 significantly decreased (**Figure 4**). After 10 days, the patient was discharged smoothly. Three months later, the patient presented with jaundice accompanied by intermittent abdominal pain, poor appetite, nausea, and vomiting. Subsequent abdominal enhanced MRI indicated a mass in the porta hepatis region, along with multiple enlarged lymph nodes around the liver, in the abdominal cavity, and retroperitoneally, suggesting recurrence (**Figures 1F, G**). Laboratory tests revealed ALT level 175 U/L, TBIL level 146 umol/L, CA19-9 level 357 U/L (**Figure 4**). The tumor compressed the biliary duct at the porta hepatis, causing obstructive jaundice, and also exerted pressure on the duodenum, resulting in upper gastrointestinal obstruction. Percutaneous transhepatic cholangial drainage was performed under ultrasound guidance to relieve the biliary obstruction and alleviate jaundice. Nasojejunal catheterization was done via endoscopy to support enteral nutrition (**Figure 1H**). Upon alleviation of jaundice and improvement in liver function, the patient declined further adjuvant therapies such as chemotherapy and voluntarily requested discharge. Subsequently, through telephone follow-up, we learned that the patient succumbed to severe malnutrition and multi-organ failure six months postoperatively.

## Discussion

ASC is a malignant tumor that comprises both AC and SCC components. It can occur in various organs throughout the body. Primary hepatic ASC, considered a subtype of ICC, is an extremely rare clinical occurrence, accounting for approximately 2%-3% of ICC cases (1, 2). Since its first description as hepatic ASC by Barr et al. (3) in 1975, there have been fewer than 100 reported cases documented internationally. Early diagnosis of primary hepatic ASC presents challenges, with most cases confirmed through postoperative pathological examination. Patients are often diagnosed at an advanced stage, making it a more aggressive and prognostically unfavorable malignancy compared to ICC (4).

The pathogenesis of primary hepatic ASC remains unclear. Most scholars suggest that it arises from the squamous metaplasia of normal bile duct cells under prolonged stimulation from chronic inflammation or viral infection, leading to malignant transformation and concurrent AC components, ultimately culminating in ASC (5, 6). In this case, the patient did not exhibit pathogenic factors such as bile duct cysts or intrahepatic bile duct stones associated with chronic inflammation, nor did they have viral hepatitis. For patients like this, it is conceivable that genetic mutations may trigger the transformation of hepatic multipotent stem cells into SCC. Further research is necessary to elucidate the underlying mechanisms of primary hepatic ASC.

Primary hepatic ASC commonly occurs in elderly patients. Most individuals present with fever, jaundice, abdominal pain, anorexia, and weight loss. Typically, there is no history of hepatitis or liver cirrhosis (7, 8). Laboratory examinations may reveal varying degrees of elevation in serum carcinoembryonic antigen (CEA) and CA19-9, while alpha-fetoprotein (AFP) levels are usually within the normal range. This characteristic is similar to ICC (9). In some cases, there may be an increase in SCC-associated antigens (4, 10–13). Due to the infrequent utilization of tumor marker tests associated with AC in patients with hepatic tumors, coupled with the low incidence rate of primary hepatic ASC, clinicians often lack awareness regarding this particular ailment. Consequently, it becomes arduous to associate this condition with its occurrence during the initial diagnosis.

Primary hepatic ASC lacks highly distinctive radiological features. In CT scans, it may manifest as an indistinct-bordered mass, occasionally accompanied by central necrosis and intrahepatic bile duct stones. Enhanced scans reveal insignificant tumor enhancement. In MRI scans, most tumors exhibit low signal intensity on T1-weighted imaging (T1WI) and high signal intensity on T2-weighted imaging (T2WI). Some cases may demonstrate high signal intensity on T1WI, possibly associated with cystic structures and central necrosis. The tumor’s diffusion is restricted, and enhanced scans show minimal tumor enhancement (14–16). In this case, the patient had normal AFP and CEA levels but a significant elevation in CA19-9. MRI showed a single tumor with irregular borders and a low signal-intensity necrotic area in the center. The contrast-enhanced scan indicated no significant enhancement, which aligns with the typical radiological features of primary hepatic ASC. However, careful differentiation from liver abscesses and ICC is necessary since these entities have been reported to be commonly mistaken and misdiagnosed as primary hepatic ASC preoperatively. PET-CT can further delineate the benign or malignant nature of the tumor and exclude systemic metastatic diseases. However, according to the literature we reviewed, ASC does not exhibit specific features on PET-CT scans. For patients highly suspected of hepatic ASC preoperatively, consideration can be given to performing a pathological biopsy to confirm the diagnosis and formulate a more scientifically rational treatment plan for the patient. Given the extreme rarity and lack of specific clinical manifestations of ASC, conventional preoperative pathological biopsy for such patients may pose certain difficulties. Therefore, preoperative diagnosis of primary hepatic ASC is extremely challenging, and in the vast majority of cases, confirmation relies on postoperative pathology and immunohistochemistry results.

Histologically, ASC comprises two cellular components: AC and SCC. AC is characterized by the formation of glands of various sizes containing intracellular and extracellular mucins. SCC, on the other hand, exhibits regular tumor cell morphology with clear boundaries, nest-like distribution, eosinophilic cytoplasm, and varying degrees of keratin pearls and intercellular bridges (17, 18). According to the prevailing diagnostic criteria established by the WHO, ASC can be definitively diagnosed when both AC and SCC components exceed 10%. In this case, well-differentiated AC accounts for approximately 40% while poorly differentiated SCC accounts for approximately 60%, meeting the diagnostic criteria for primary hepatic ASC. Immunohistochemically, different types of cytokeratins (CKs) are expressed in hepatocytes and biliary epithelial cells. In immunohistochemical staining of hepatocellular carcinoma (HCC), CK20 typically shows positive reactivity, while CK7 and CK19 show negative reactivity. However, in ICC, CK20 is mostly negative, while CK7 and CK19 are positive. As primary hepatic ASC represents a subtype of ICC, its immunohistochemical profile generally aligns with the characteristics of ICC: CK20(-), CK7(+), CK19(+), and the presence of SCC-associated tumor markers p63/p40(+) can further aid in the accurate diagnosis (9). The postoperative pathological and immunohistochemical results in this case largely conform to the aforementioned characteristics.

Curative surgical resection stands as the primary therapeutic modality for non-metastatic primary hepatic ASC. The surgical approach follows the treatment principles of ICC, encompassing the excision of the affected liver lobe coupled with clearance of the hepatic hilum lymph nodes. The specific surgical technique and extent of lymph node clearance are determined based on lesion location, local vascular, biliary, lymphatic infiltration, and dissemination. Performing curative resection according to tumor location, along with thorough clearance of the hepatic hilum lymph nodes, can maximize patient prognosis. We believe that an anatomical liver lobectomy, with a margin width exceeding 1cm, and comprehensive clearance of the hepatic hilum lymph nodes can confer substantial benefits to patients by prolonging tumor recurrence and improving overall prognosis.

Primary hepatic ASC exhibits a poor prognosis. Takahashi et al. (19) employed DNA ploidy quantitative analysis techniques, indicating that the SCC component demonstrates a more malignant biological behavior closely associated with rapid tumor growth and invasive clinical characteristics. Hence, there is reason to believe that a higher proportion of SCC component corresponds to increased malignancy. Based on previous case reports, the average survival time for such cases falls short of 1 year. While curative surgical resection stands as the foremost therapeutic approach for non-metastatic primary hepatic ASC, even in most cases where curative resection is performed, there is still a propensity for early tumor recurrence, lymph node metastasis, and even distant metastasis. Solely relying on surgical intervention yields a less optimistic prognosis (16, 20). Research has shown that patients with primary hepatic ASC who undergo adjuvant chemotherapy experience longer survival (median survival: 15 months vs. 6 months) (9). Studies conducted by Kang (11) and Demir (21) also indicate a significant extension in survival for patients with primary hepatic ASC who receive adjuvant chemotherapy, with the highest reported survival reaching 8 years. Watanabe et al. (22) summarized treatment and prognosis data for 71 patients with primary hepatic ASC from PubMed. Among the 48 patients who solely underwent surgical treatment, 39 (92.9%) died within 12 months postoperatively. In contrast, Among the 7 patients who received adjuvant chemotherapy after surgery, 5 patients did not experience tumor recurrence during the 8-15 months follow-up period. It is worth mentioning that one patient missed the opportunity for curative surgery at initial diagnosis but underwent surgical resection following systemic chemotherapy with tegafur/gimeracil/oteracil (S-1) and transcatheter hepatic arterial injection (TAI) of cisplatin. The patient then received adjuvant cisplatin chemotherapy for 6 months, resulting in a favorable prognosis with no tumor recurrence during the 6-month follow-up period. These studies collectively affirm the effectiveness of adjuvant chemotherapy. Watanabe’s research (22) suggests that combination therapy with systemic chemotherapy and hepatic arterial infusion cisplatin demonstrates favorable efficacy for unresectable primary hepatic ASC patients, and may even create opportunities for curative resection. This indirectly confirms the efficacy of HAIC treatment. The aim of using HAIC is to locally deliver higher concentrations of chemotherapeutic agents and alleviate systemic toxicity. We believe that HAIC treatment can be considered for patients with significant local tumor invasion, high-risk recurrence postoperatively, and those unable to tolerate systemic chemotherapy. Referring to the chemotherapy regimen of ICC, the combination of gemcitabine and cisplatin is considered the most effective first-line treatment (23), which may also yield good results for hepatic ASC patients. Consequently, comprehensive treatment based on surgery has gradually emerged as a novel direction in recent research. In the future, the combined treatment approach of surgery with adjuvant chemotherapy holds the potential to become the optimal choice for treating primary hepatic ASC. Moreover, the conversion therapy model, chemotherapy-surgery-postoperative chemotherapy, may also offer a glimmer of hope for survival among advanced-stage patients who have missed the opportunity for surgical intervention.

In this case, the patient declined any adjuvant therapy postoperatively, including chemotherapy and radiotherapy. The prognosis was poor, with tumor recurrence occurring only 3 months after surgery, accompanied by intrahepatic and intra-abdominal lymph node metastasis. The patient passed away 6 months after the surgery, greatly deviating from our expectations and serving as a profound reminder of the postoperative significance of adjuvant chemotherapy. Perhaps mere surgical treatment cannot achieve satisfactory efficacy for hepatic ASC with lymph node metastasis. Active cooperation with adjuvant chemotherapy postoperatively is essential to further improve the prognosis of such patients. The patient’s refusal of further adjuvant chemotherapy postoperatively delayed the optimal treatment window, resulting in an unsatisfactory prognosis, which we deeply regret. This is a limitation in our case and calls for the reflection of experiences and lessons learned from this treatment process. We cannot predict whether neoadjuvant chemotherapy would yield better therapeutic effects preoperatively, as it is a relatively unfamiliar field in the treatment of hepatic ASC. Additionally, not every patient is highly sensitive to chemotherapy, and disease progression could lead to the loss of the opportunity for curative surgery. For patients with the opportunity for R0 resection, we recommend early surgical intervention combined with adjuvant chemotherapy as the optimal treatment choice for such cases.

For advanced primary hepatic ASC patients who have lost the opportunity for surgery and those who experience postoperative recurrence, ongoing attempts are being made to explore the efficacy of molecular targeted therapy and immunotherapy. In recent years, targeted drugs and immunotherapy have shown good efficacy in patients with advanced ICC (24). As a clinical subtype of ICC, hepatic ASC shares many similarities with ICC. We have reason to believe that with in-depth research on the molecular mechanisms, gene sequencing technology, and therapeutic targets of ASC, molecular targeted therapy and immunotherapy may become breakthroughs in the future treatment of ASC. In the case of poor prognosis in advanced-stage hepatic ASC patients, further exploration is warranted regarding the utilization of local treatment, targeted therapy, immunotherapy, and their combination. Additionally, considering the higher sensitivity of the SCC component compared to AC towards radiation therapy, which has shown good efficacy in treating SCC in other sites, some scholars advocate for implementing radiotherapy in hepatic ASC cases with a higher proportion of SCC. However, due to limited sample size and experience, the effectiveness of these treatment modalities requires further confirmation through additional accumulation of cases. ASC exhibits severe malignant biological behavior, characterized by highly invasive and rapid growth mechanisms that remain unclear. Despite aggressive treatments, overall therapeutic outcomes for most patients remain unsatisfactory, possibly due to tumor heterogeneity and the malignant features of SCC, among other influencing factors. Improving early diagnosis rates would contribute to enhancing patient prognosis.

## Conclusion

In light of the aforementioned exposition, primary hepatic ASC is an extremely rare and highly malignant liver tumor. It exhibits rapid disease progression and presents significant challenges in preoperative diagnosis. Most patients are diagnosed at advanced stages, leading to poor prognosis and high mortality rates. We believe that an integrated treatment approach combining surgery with adjuvant chemotherapy can effectively prolong patient survival.

## Data availability statement

The original contributions presented in the study are included in the article/supplementary material, further inquiries can be directed to the corresponding author.

## Ethics statement

Written informed consent was obtained from the individual(s) for the publication of any potentially identifiable images or data included in this article.

## Author contributions

HA: Writing – original draft. TG: Writing – review & editing. YM: Writing – review & editing. GM: Writing – review & editing. JZ: Writing – review & editing. XZ: Writing – review & editing.
